# Synthesis and electrochemical properties of 3,4,5-tris(chlorophenyl)-1,2-diphosphaferrocenes

**DOI:** 10.3762/bjoc.18.139

**Published:** 2022-09-27

**Authors:** Almaz A Zagidullin, Farida F Akhmatkhanova, Mikhail N Khrizanforov, Robert R Fayzullin, Tatiana P Gerasimova, Ilya A Bezkishko, Vasili A Miluykov

**Affiliations:** 1 Arbuzov Institute of Organic and Physical Chemistry, FRC Kazan Scientific Center, Russian Academy of Sciences, 8 Arbuzov Street, 420088 Kazan, Russian Federation

**Keywords:** cyclopropenyl bromide, electrochemical properties, phosphacyclopentadienide anion, phosphaferrocene, phosphonium salt, phosphorus heterocycle

## Abstract

A novel representative of sodium 3,4,5-triaryl-1,2-diphosphacyclopentadienide containing a chloro substituent in the *meta*-position of the aryl groups was obtained with a high yield based on the reaction of tributyl(1,2,3-triarylcyclopropenyl)phosphonium bromide and sodium polyphosphides. Further reaction of sodium 3,4,5-tris(3-chlorophenyl)-1,2-diphosphacyclopentadienide with [FeCp(η^6^-C_6_H_5_CH_3_)][PF_6_] complex gives a new 3,4,5-tris(3-chlorophenyl)-1,2-diphosphaferrocene. The electrochemical properties of 3,4,5-tris(3-chlorophenyl)-1,2-diphosphaferrocene were studied and compared to 3,4,5-tris(4-chlorophenyl)-1,2-diphosphaferrocene. It was found that the position of the chlorine atom on the aryl fragment has an influence on the reduction potential of 1,2-diphosphaferrocenes, while the oxidation potentials do not change.

## Introduction

Among the various heterometallocenes reported to date, phosphaferrocenes are by far the most investigated because of their structural and electronic features [1–2] and remain the objects of growing interest in the fields of coordination chemistry [3–5] and asymmetric catalysis [6–7]. Due to the sp^2^-hybridization of the phosphorus atom, phosphaferrocenes are commonly regarded as phosphorus ligands with weaker σ-donor character than classical tertiary phosphines and stronger π-acceptor properties closer to phosphites P(OR)_3_ [8–9]. Since the P atom in phosphaferrocenes retains an electron lone pair, phosphaferrocenes have been used as P-donor ligands [10–12] as well as nucleophilic catalysts [13–14]. Recently, the pentaphosphaferrocene Cp*Fe(η^5^-P_5_) has been used as a mediator in the synthesis of asymmetric phosphines starting from white phosphorus [15]. Moreover, the presence of the lone pair of the P atom opens the route to polynuclear complexes [16–18] and coordination polymers [19–21] with the mixed σ-/π-coordination mode, which is not typical for classical ferrocene species.

Various effective synthetic approaches were developed for 1-mono- [22–24], 1,2,3-tri- [25–27], 1,2,4-tri- [28–30], and pentaphosphaferrocenes [31–33], whereby the chemistry of these compounds is most investigated and well represented at present time. In contrast, very limited data are available concerning 1,2-diphosphaferrocenes due to the absence of simple and effective synthetic routes [34–36]. Recently, we have reported a convenient synthesis of 3,4,5-triaryl-1,2-diphosphaferrocenes with various substituents at the *para-*positions of aryl groups [37]. Based on this method, herein we report on the complete multistep synthesis of new sodium 3,4,5-tris(3-chlorophenyl)-1,2-diphosphacyclopentadienide and corresponding 1,2-diphosphaferrocene with *meta-*chlorophenyl substituents and the influence of the position of the Cl atom on aryl moiety on the electrochemical properties.

## Results and Discussion

### Synthesis of tris(chlorophenyl)cyclopropenyl bromides and derivatives

Cyclopropenium (cyclopropenylium) ions have always attracted attention of the synthetic chemists because of the unique combination of stability and reactivity [38–40]. The synthesis of corresponding 1,2,3-cyclopropenium bromides was realized by a classical approach: combination of C_1_ and C_2_ building blocks, i.e., the addition of a carbene species to a triple bond of diarylacetylene, followed by treatment of the produced cyclopropene with HBr to convert it to the corresponding cyclopropenylium cation. Using this approach, tris(4-chlorophenyl)- and tris(3-chlorophenyl)cyclopropenyl bromides were prepared for the first time. The advantage of this approach is the possibility of synthesis of substituted diarylacetylenes, the corresponding substituted benzal chlorides, and triarylcyclopropenyl bromides from one starting aryl aldehyde.

**Scheme 1:**
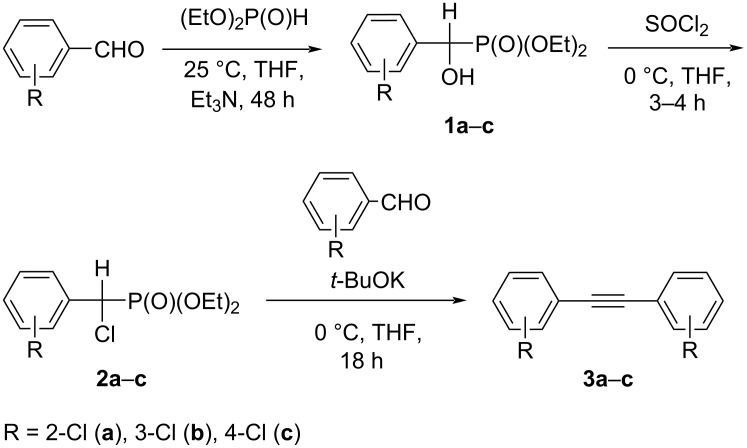
Synthesis of bis(chlorophenyl)acetylenes **3**.

Diethyl phosphite was allowed to react with appropriately substituted benzaldehydes in THF for 48 hours at 25 °C to afford diethyl (hydroxy(aryl)methyl)phosphonates **1**, which were detected by ^31^P NMR spectroscopy in THF (21.4 ppm for **1a**, 21.0 ppm for **1b**, and 21.5 ppm for **1c**). Further, reaction mixtures with compounds **1** were treated with SOCl_2_ for 3–4 h at 0 °C and converted to chloro derivatives **2**. In the next step, compounds **2** and starting substituted benzaldehydes were subsequently treated with 2 equiv of potassium *tert*-butoxide in THF for 18 hours at room temperature to afford substituted diarylacetylenes **3**. Based on this reaction, the desired compounds **3** were prepared from 2-chloro-, 3-chloro-, and 4-chlorophenyl aldehydes, respectively, in 3 steps in 10–53% yield (10% for **3a**, 48% for **3b**, 53% for **3c**, Scheme 1). This method is an alternative way to different transition metal-catalyzed cross-coupling reactions broadly used for the preparation of different diarylacetylenes and, rarely, bis(chlorophenyl)acetylenes.

**Scheme 2:**
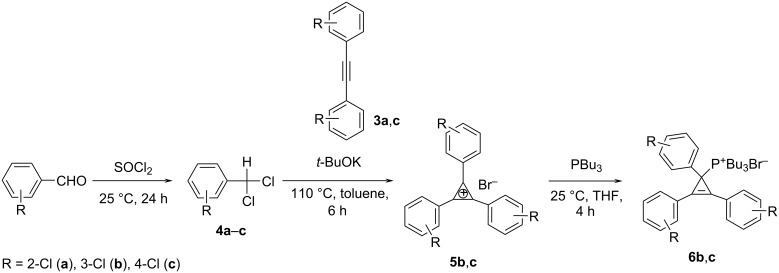
Synthesis of 1,2,3-tris(chlorophenyl)cyclopropenylium bromides **5** and tributyl(1,2,3-tris(chlorophenyl)cyclopropenyl)phosphonium bromides **6**.

Next, starting substituted benzaldehydes were treated with an excess of SOCl_2_ for 24 h at 25 °C. Corresponding substituted benzal chlorides **4** were distilled at reduced pressure to give pure compounds. In a final step, we used the above mentioned approach of combining the C_1_ and C_2_ building blocks and found that chloroarylcarbenes, generated from the corresponding benzal chlorides **4b**,**c** under the action of potassium *tert*-butoxide, reacted with 1,2-bis(chlorophenyl)ethynes **3b**,**c** to form triarylcyclopropenylium salts **5b**,**c** in 22 and 15% yield (Scheme 2). Unfortunately, it was not possible to synthesize tris(2-chlorophenyl)cyclopropenylium bromide **5a** using this method.

**Figure 1:**
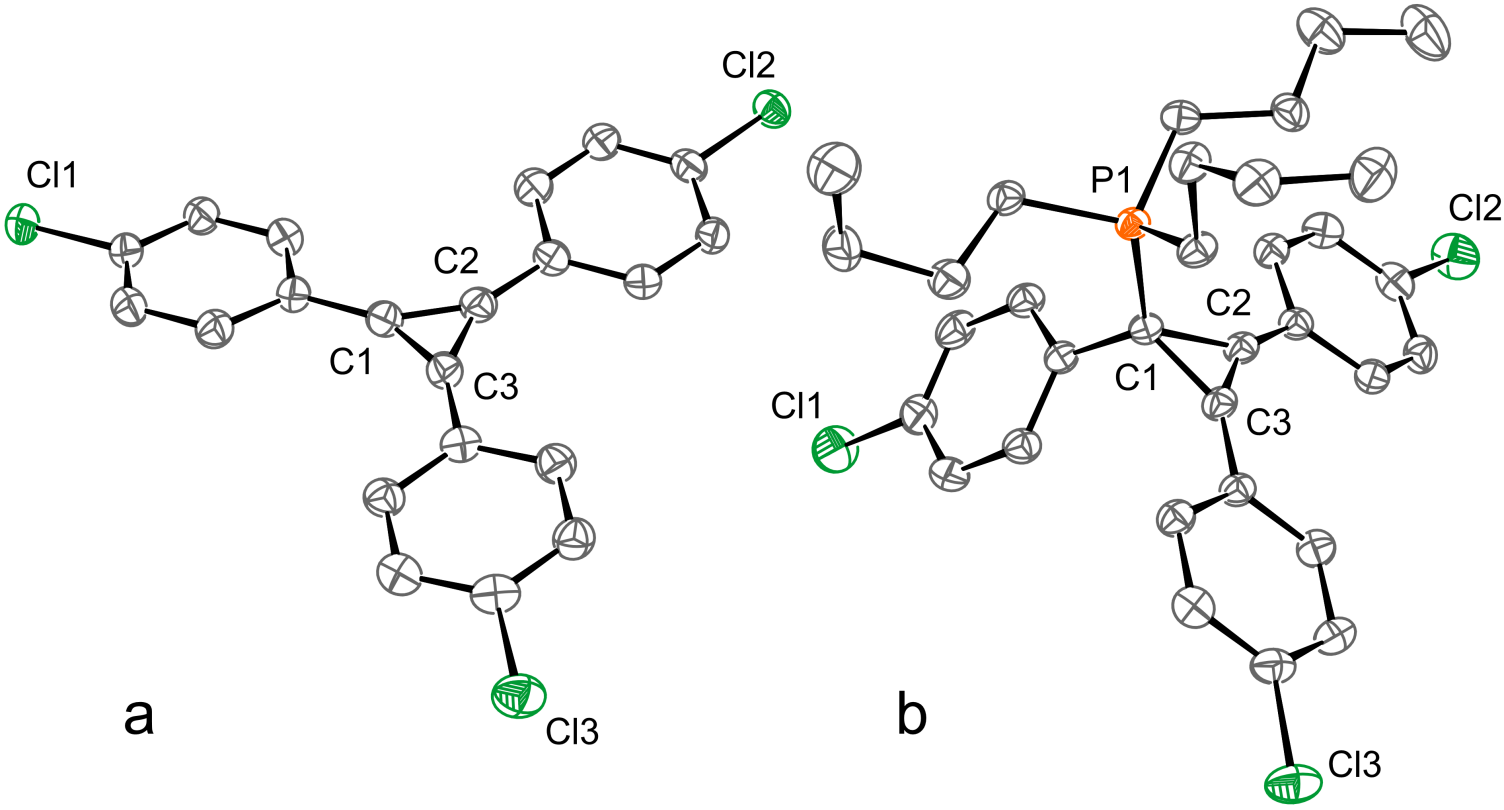
ORTEP representations for cations **5c** (a) and **6c** (b) at the 50% probability level. Bromide anion and co-crystallized solvent molecules are omitted for clarity. For **6c**, only one of two symmetry-independent molecules is shown. Selected interatomic distances (Å): C1–C2 1.387(7), C1–C3 1.372(7), C2–C3 1.380(7) for **5c**; C1–C2 1.521(5), C1–C3 1.521(5), C2–C3 1.298(5), P1–C1 1.837(3) for **6c**. Deposition numbers 2176393 for **5c** and 2176394 for **6c** contain the supplementary crystallographic data for this paper [41].

The structures of **3**–**5** were confirmed by ^1^H and ^13^C NMR as well as IR spectroscopic methods and, for **5c**, single-crystal X-ray crystallography (Figure 1a). The ^13^C NMR signals of the cationic carbon atoms of the three-membered ring appeared at about 145 ppm. Besides, the ^1^H NMR spectra of **5** were unremarkable and consistent with the suggested formulas.

As a next step, we synthesized a series of tributyl(1,2,3-triarylcyclopropenyl)phosphonium bromides **6** containing a Cl substituent in the *meta-* or *para*-position of each aryl group. This was done by reaction of appropriate 1,2,3-triarylcyclopropenylium bromides **5** with PBu_3_ at 25 °C in THF in 34 and 39% yield (Scheme 2). The structures of **6** were confirmed by ^31^P, ^1^H, and ^13^C NMR spectroscopy. The ^31^P{^1^H} NMR spectra of phosphonium bromides **6** showed a singlet at about 40 ppm, which is typical for phosphonium salts. The ^13^C{^1^H} NMR spectra consisted of a doublet at about 20 ppm, corresponding to the carbon atom C1, which is characteristic for the sp^3^-hybridized carbon atom, with a coupling constant of ^1^*J*_CP_ ≈ 45 Hz. Additionally, the structure of **6c** in the crystal was confirmed by X-ray crystallography (Figure 1b).

### Synthesis, structure, and electrochemical properties of 3,4,5-tris(chlorophenyl)-1,2-diphosphaferrocenes

**Scheme 3:**
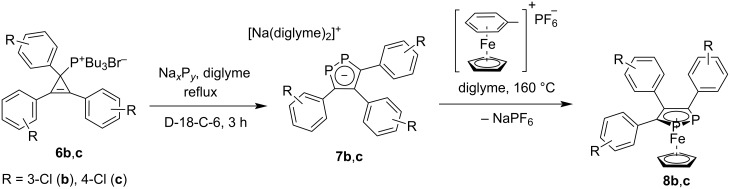
Synthesis of 3,4,5-tris(chlorophenyl)-1,2-diphosphacyclopentadienides **7** and 3,4,5-tris(chlorophenyl)-1,2-diphosphaferrocenes **8**.

The obtained phosphonium salts **6** were treated with a mixture of sodium polyphosphides of the type Na*_x_*P*_y_* (obtained in situ from sodium metal and white phosphorus P_4_), containing mainly NaP_5_ and Na_3_P_7_ [42]), resulting in sodium 3,4,5-tris(chlorophenyl)-1,2-diphosphacyclopentadienides **7** in good yields (60 and 63%, Scheme 3). This reaction allowed a selective and controllable conversion of Na*_x_*P*_y_* to the 1,2-diphospholide anion, in which two new C−P bonds could selectively be formed [43–44]. The obtained sodium 3,4,5-triaryl-1,2-diphospholides **7** were isolated in good purity from the reaction mixture by filtration and further washing with a mixture of THF/*n*-hexane. The ^31^P{^1^H} NMR spectra of **7** showed a singlet at about 200 ppm, which is typical for sodium 1,2-diphospholides (^31^P{^1^H} in THF: 201 ppm for **7b** and 198 ppm for **7c**). Further, the ^13^C{^1^H} NMR spectra of **7** showed two multiplets at about 147 and 160 ppm for the heteroaromatic P_2_C_3_ ring backbone.

Recently, we have reported a convenient method for the preparation of 1,2-diphosphaferrocenes [37] and 1,2,3-triphosphaferrocenes [25] with various substituents at *para-*positions of aryl groups. Using this approach, sodium bis(diglyme) 3,4,5-tris(3-chlorophenyl)-1,2-diphosphacyclopentadienide (**7b**) was treated in a 1:1 ratio with [FeCp(η^6^-C_6_H_5_CH_3_)][PF_6_] at 160 °C in diglyme. Evaporation of diglyme at reduced pressure and extraction of the product with toluene, followed by filtration through silica, resulted in 3,4,5-tris(3-chlorophenyl)-1,2-diphosphaferrocene (**8b**) in 68% yield and high purity (Scheme 3).

The structure of 3,4,5-triaryl-1,2-diphosphaferrocene **8b** was confirmed by ^31^P, ^1^H, and ^13^C NMR spectroscopy. The ^31^P{^1^H} NMR spectrum of **8b** showed a singlet at about −10 ppm, shifted upfield by about 210 ppm in comparison to the starting 1,2-diphospholide anion **7b**. In the ^1^H NMR spectra, characteristic signals of the cyclopentadienyl ring (4.61 ppm) and ClC_6_H_4_ substituents (6.88–7.42 ppm) were observed. The ^13^C{^1^H} NMR spectrum of **8b** showed pseudotriplets at about 106 ppm and 117 ppm for the carbon atoms of the P_2_C_3_ ring and a singlet at about 75 ppm for the cyclopentadienyl ring.

**Figure 2:**
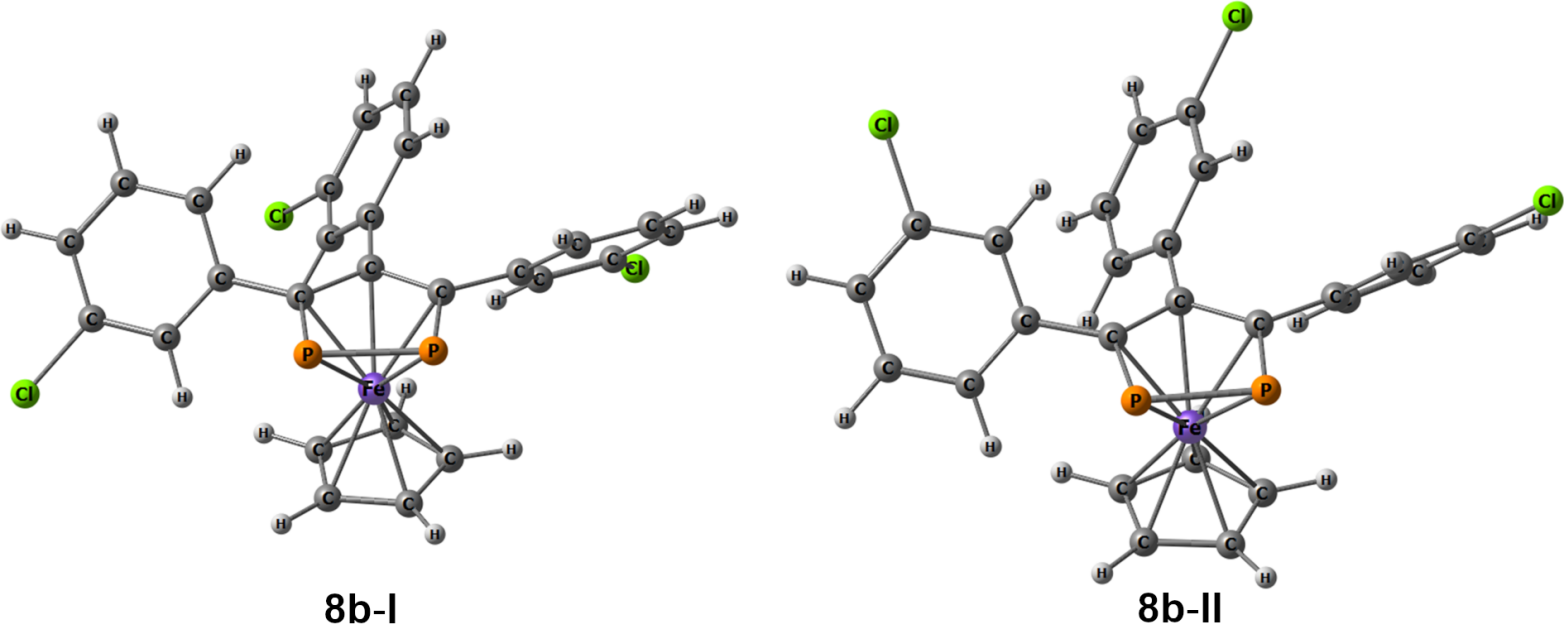
Considered conformations of **8b-I** and **8b-II**.

Quantum chemically, two possible conformations of **8b** were considered, **8b**-**I** and **8b**-**II** (Figure 2). Similar to a previous report on **8c** [37], both **8b**-**I** and **8b**-**II** adopted an almost eclipsed conformation during optimization. Computations predicted slightly lower energy (1 kcal⋅mol^−1^) for conformation **8b-I**, with Cl oriented towards the Fe atom. According to the computations, **8b-I** is also slightly more advantageous compared to **8c** with the same energy difference.

**Figure 3:**
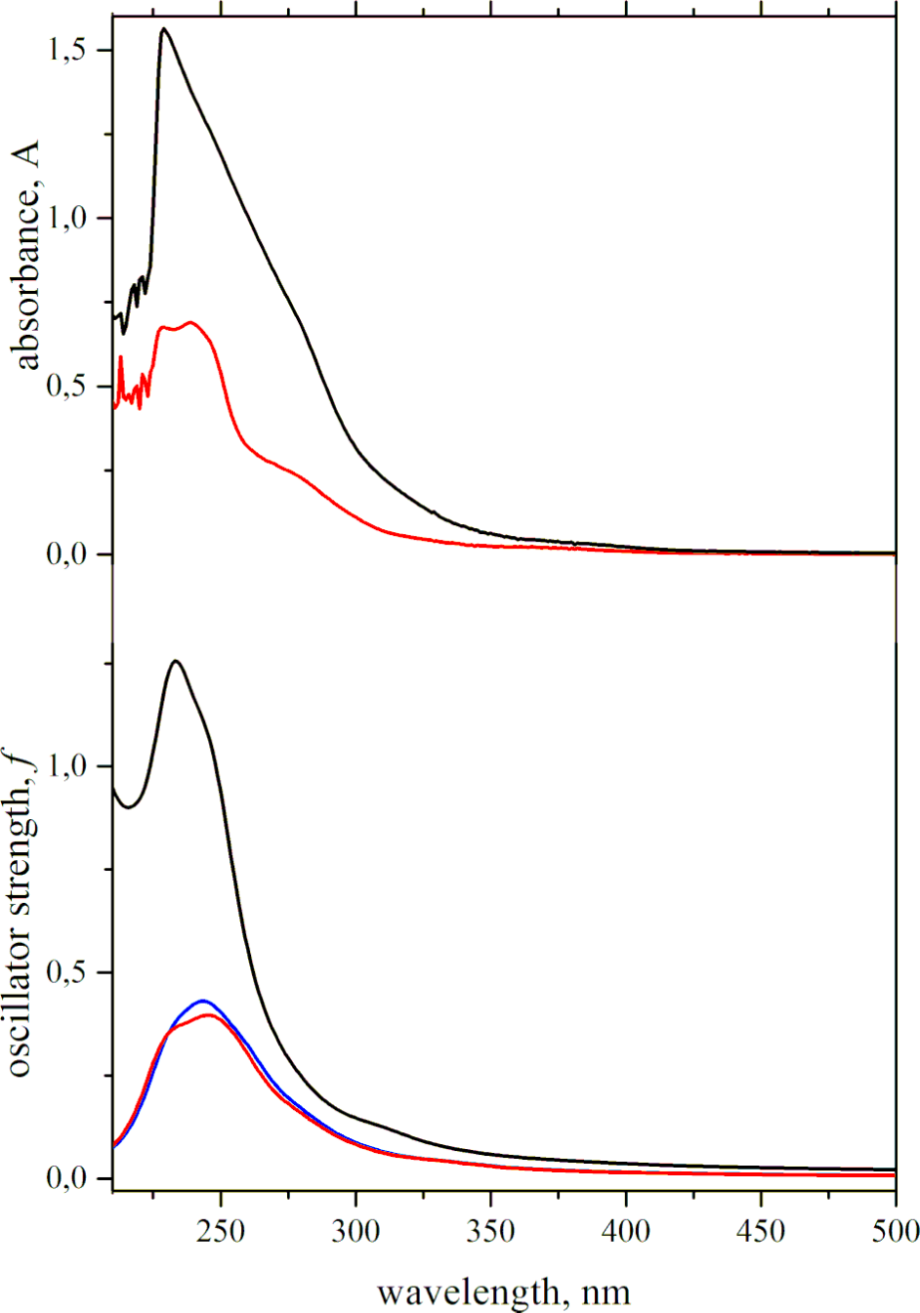
Top: experimental UV–vis spectra of **8с** (black) and **8b** (red). Bottom: broadened calculated UV–vis spectra of **8c** (black), **8b-I** (red), and **8b**-**II** (blue). Experimental UV–vis spectra of **8с** republished with permission of Royal Society of Chemistry from [37] (“Synthesis, structure and electrochemical properties of 3,4,5-triaryl-1,2-diphosphaferocenes” by I. A. Bezkishko et al., *Inorg. Chem. Front.*, vol. 9, Issue 11, © 2022); permission conveyed through Copyright Clearance Center, Inc. This content is not subject to CC BY 4.0.

**Figure 4:**
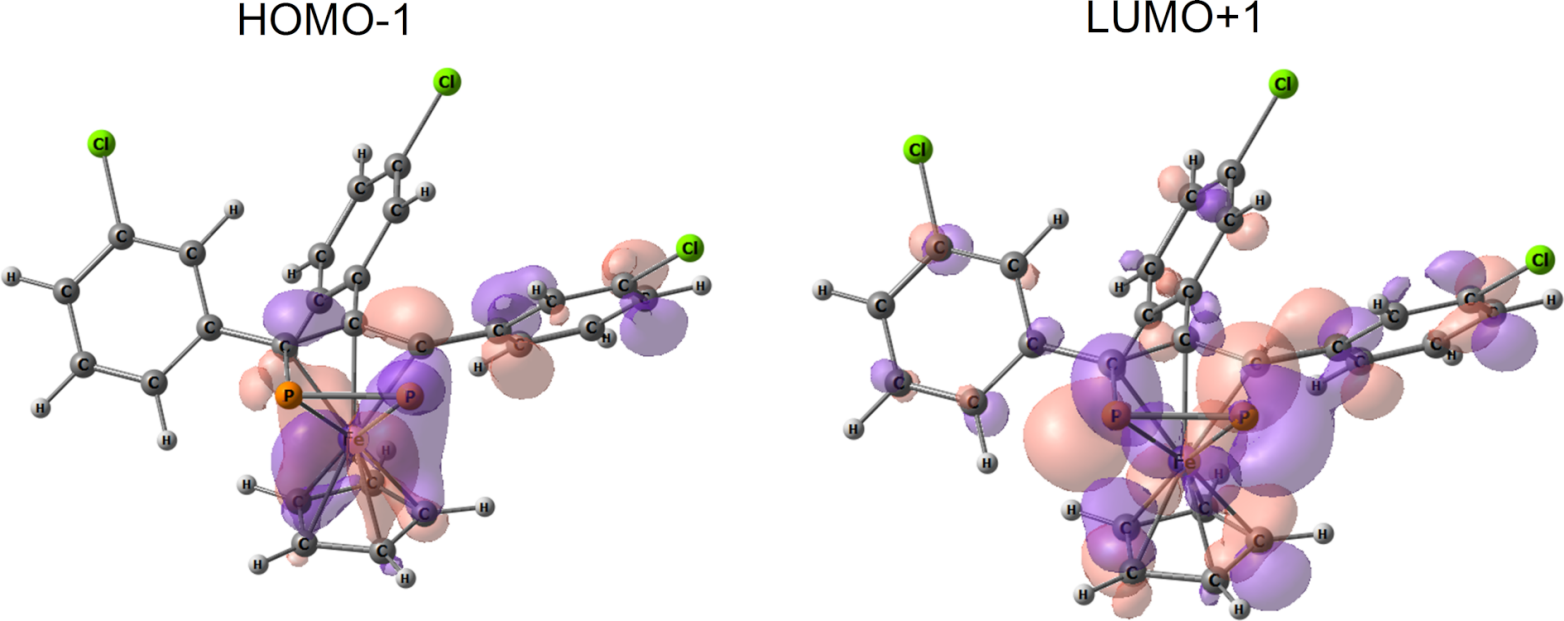
Frontier orbitals of **8b**-**II** contributing to absorption bands at about 380 nm.

The experimental UV–vis spectra of **8b** and **8c** in CH_2_Cl_2_ were similar and contained bands at about 280, 320, and 380 nm. The bands at 280 and 320 nm were more intense in the spectrum of **8c**, which is in full agreement with quantum chemical predictions (Figure 3). According to the computations, **8b**-**I** and **8b**-**II** demonstrated almost the same absorption. The bands at about 250 and 280 nm were caused by π–π* transitions. The dominating transition contributing to the lowest-energy absorption (380 nm) was the one corresponding to a transition between HOMO−1 and LUMO+1 (Figure 4). Both orbitals were localized mostly at the P_2_C_3_–Fe–Cp moiety, and the former was also contributed to by atomic orbitals of the aryl ring in the 4-position. Similar to **8c**, the atomic orbitals of the Cl atoms in **8b** practically did not participate in the frontier orbitals, which explained the similarity of the low-energy range of the experimental spectra.

**Figure 5:**
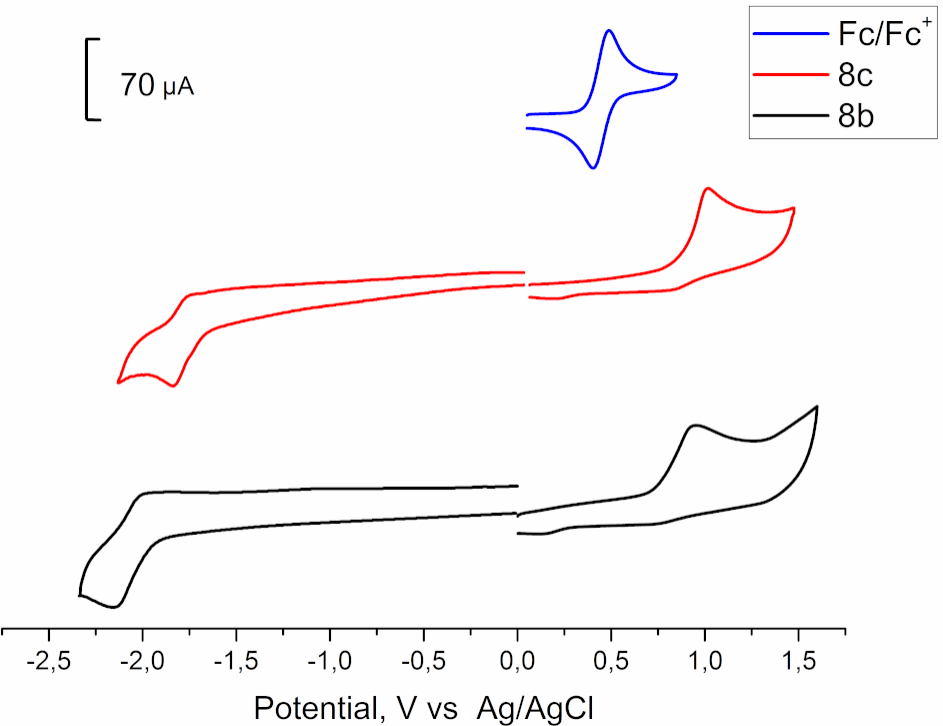
Cyclic voltammograms of 3,4,5-triaryl-1,2-diphosphaferrocenes **8b** and **8c** in CH_3_CN on glassy carbon electrode (0.5 mM complex). Potentials vs Ag/AgCl. Scan rate = 100 mV⋅s^−1^, room temperature. Cyclic voltammogram of **8c** republished with permission of Royal Society of Chemistry from [37] (“Synthesis, structure and electrochemical properties of 3,4,5-triaryl-1,2-diphosphaferocenes” by I. A. Bezkishko et al., *Inorg. Chem. Front.*, vol. 9, Issue 11, © 2022); permission conveyed through Copyright Clearance Center, Inc. This content is not subject to CC BY 4.0.

The electrochemical properties of 1,2-diphosphaferrocene **8b** were studied by cyclic voltammetry and compared to data for **8c** (Table 1 and Figure 5). During oxidation, there were no noticeable differences between **8b** and **8c**. The oxidation potentials of **8b** and **8c** were shifted to the positive region relative to pure ferrocene by 0.48–0.53 V. This, in turn, indicated that the chlorine atoms in the η^5^-P_2_C_3_R_3_ fragment did not significantly affect the HOMO energy of 1,2-diphosphaferrocenes **8**. The number of phosphorus atoms in cyclopentadiene had a much greater effect on the shifts of the Fe^II^/Fe^III^ oxidation potential. As shown earlier, an increase in the number of phosphorus atoms led to the irreversible oxidation of phosphaferrocenes containing an unsubstituted Cp ring. It was shown that Cp*Fe(η^5^-P_5_) was irreversibly oxidized at a potential of 0.57 V relative to Fс/Fс^+^, and the presence of even five phosphorus atoms makes an insignificant contribution to the HOMO energy level [45]. The situation changed fundamentally when both rings were replaced with phosphacyclopentadienyl ligands. Related diphosphacyclobutadiene complexes Fe(η^4^-P_2_C_2_R_2_)_2_ were oxidized much more cathodically (negative by 1.7–2.0 V) [46–47], which indicated a significant contribution of the phosphacyclopentadienyl ligands to the iron atomic orbitals. Of course the structures of **8** and diphosphacyclobutadiene complexes are not isolobal, but it would be interesting to study electrochemical properties of Fe(η^5^-P_2_C_3_R_3_)_2_ complexes in the future.

**Table 1:** Electrochemical data for the redox properties of 3,4,5-triaryl-1,2-diphosphaferrocenes **8b** and **8c**.

compound	*E*_ox_^1^ (V) vs Ag/AgCl	*E*_red_^1^ (V)	^1^*E*_HOMO_ (eV)	^1^*E*_LUMO_ (eV)	gap (eV)

ferrocene [48]	0.48	−3.19^a^	−4.79^a^	−1.61^a^	3.18^a^
FeCp(η^5^-P_2_C_3_R_3_) (R = 3-Cl-C_6_H_4_, **8b**)	0.96	−2.15	−5.28	−2.2	2.84
FeCp(η^5^-P_2_C_3_R_3_) (R = 4-Cl-C_6_H_4_, **8c**) [37]	1.01	−1.83	−5.36	−2.48	2.88
Cp*Fe(η^5^-P_5_) [45]	1.12^b^	−1.55^b^	−5.47^b^	−2.80^b^	2.57^b^

^a^Conditions: −50 °C, glassy carbon working electrode, Ag/AgCl reference electrode, *c* 0.5 mM, Bu_4_NBF_4_, DMF, 100 mV⋅s^−1^. ^b^Conditions: −13 °C, Pt working electrode, Ag/AgCl reference electrode (recalculated from Fc/Fc^+^), *c* 0.5 mM, Bu_4_NPF_6_, CH_2_Cl_2_, 500 mV⋅s^−1^.

For reduction, the electrochemical properties changed more noticeable since the contribution to the LUMO came from the cyclopentadiene fragments. For 1,2-diphosphaferrocene **8b**, the reduction potential was positively shifted by 0.32 V as compared to **8c**. It should be noted that an increase of phosphorus atoms' number in phosphaferrocenes leads to a greater positive potential, which in turn leads to the formation of dimers, which was shown for pentaphosphaferrocene Cp*Fe(η^5^-P_5_) [49] and the corresponding Sm complexes [50].

## Conclusion

In summary, a series of bis(сhlorophenyl)acetylenes **3**, substituted benzal chlorides **4**, and tris(chlorophenyl)cyclopropenylium bromides **5** were synthesized starting from corresponding chloro-substituted benzaldehydes. We found that the reaction of tributyl(1,2,3-tris(chlorophenyl)cyclopropenyl)phosphonium bromides **6** with sodium polyphosphides can be successfully used for the preparation of sodium 3,4,5-tris(chlorophenyl)-1,2-diphosphacyclopentadienides **7**. A facile synthesis of 3,4,5-tris(3-chlorophenyl)-1,2-diphosphaferrocene (**8b**) from sodium bis(diglyme) 3,4,5-tris(3-chlorophenyl)-1,2-diphosphacyclopentadienide (**7b**) and [FeCp(η^6^-C_6_H_5_CH_3_)][PF_6_] is described. The structure of **8b** was studied using experimental NMR, UV–vis, and electrochemical analyses as well as theoretical studies. The *meta*- and *para*-substitution of the Cl atoms in the aryl fragments did not significantly effect the oxidation potentials of 1,2-diphosphaferrocenes **8**, while the reduction potential of **8b** was shifted by 0.33 V to a more negative region as compared to **8c**.

## Supporting Information

File 1 Experimental procedures and characterization data of synthesized compounds.
