# Supplementary material for: Synthesis and electrochemical properties of 3,4,5-tris(chlorophenyl)-1,2-diphosphaferrocenes
Source: Beilstein J Org Chem. 2022 Sep 27;18:1338–45. doi: 10.3762/bjoc.18.139 (PMC9531555; doi:10.3762/bjoc.18.139)
Supplement: File 1 — Experimental procedures and characterization data of synthesized compounds. [file Beilstein_J_Org_Chem-18-1338-s001.pdf]

BEILSTEIN JOURNAL OF ORGANIC CHEMISTRY

Supporting Information

for

Synthesis and electrochemical properties of 3,4,5-tris(chlorophenyl)-1,2-diphosphaferrocenes

Almaz A. Zagidullin, Farida F. Akhmatkhanova, Mikhail N. Khrizanforov, Robert R. Fayzullin, Tatiana P. Gerasimova, Ilya A. Bezkishko and Vasili A. Miluykov

Beilstein J. Org. Chem. **2022,** *18,* 1338–1345. doi:10.3762/bjoc.18.139

Experimental procedures and characterization data of synthesized compounds

License and Terms: This is a supporting information file under the terms of the Creative Commons Attribution License (https://creativecommons.org/licenses/by/4.0). Please note that the reuse, redistribution and reproduction in particular requires that the author(s) and source are credited and that individual graphics may be subject to special legal provisions.
The license is subject to the *Beilstein Journal of Organic Chemistry* terms and conditions: (https://www.beilstein-journals.org/bjoc/terms)

Experimental

General

All reactions and manipulations were carried out under dry pure N_2 using standard Schlenk apparatus. All solvents were distilled from sodium and benzophenone and stored under N_2 before use. The NMR spectra were recorded on a Bruker MSL-400 (^{1}H 400 MHz, ^{31}P 161.7 MHz, ^{13}C 100.6 MHz). $SiMe_4$ was used as internal reference for ^{1}H and ^{13}C NMR chemical shifts, and 85% H_3PO_4 as external reference for ^{31}P. IR spectra were recorded on a Bruker Vector-22 spectrometer. Elemental analyses were carried out at the microanalysis laboratory of the Arbuzov Institute of Organic and Physical Chemistry, Russian Academy of Sciences.

UV–vis experiments

Electronic absorption (UV–vis) spectra were recorded at room temperature on a Perkin-Elmer Lambda 35 spectrometer using 10 mm quartz cells. Absorption spectra were registered with a scan speed of 480 nm/min, using a spectral width of 1 nm. All samples were prepared as solutions in dichloromethane with the concentrations 10^{-5} mol·L^{-1}.

DFT calculations

All calculations were performed with the Gaussian 16 suite of programs [1]. The hybrid PBE0 functional [2] and the Ahlrichs' triple-ζ def-TZVP AO basis set [3] were used for optimization of all structures. In all geometry optimizations, the D3 approach [4] was applied to describe the London dispersion interactions as implemented in the Gaussian 16 program. Time-dependent density functional theory (TDDFT) with the use of long-range-corrected CAM-B3LYP functional [5] has been employed to compute the vertical

excitation energy (i.e., absorption wavelength) and oscillator strength for the ground-state optimized geometries.

Electrochemical measurements

As described in Reference [6], the electrochemical measurements were conducted with a BASi Epsilon EClipse electrochemical analyzer (USA). The program concerned Epsilon-EC-USB-V200 waves. A conventional three-electrode system was used with glassy carbon (GC) or carbon paste electrode (CPE) solutions for powder samples as the working electrode, the Ag/AgCl (0.01 M) electrode as the reference electrode, and a Pt wire as the counter electrode. 0.1 M Et_4NBF_4 was used as the supporting electrolyte to determine the current–voltage characteristics.

To study the powder samples, a modified CPE working electrode was used, which was prepared as follows: the carbon particles/phosphonium salt dodecyl(tri-*tert*-butyl)phosphonium tetrafluoroborate composite electrode was prepared by grinding a mixture of graphite powder and phosphonium salt with a 70:30, w/w ratio in a mortar giving a homogeneous mass [7]. A modified electrode was also devised in a similar manner except that a portion (≈5%) of the graphite powder was replaced by the investigated compound under study. As a result, a portion of the resulting paste was packed firmly into the Teflon holder cavity (3 mm in diameter).

Single-crystal X-ray diffraction

The X-ray diffraction data for the single crystals **5c** and **6c** were collected on a Bruker D8 QUEST diffractometer with a PHOTON III area detector and an IμS DIAMOND microfocus X-ray tube using Mo *K*α (0.71073 Å) radiation. The diffractometer was equipped with an Oxford Cryostream LT device for low-temperature experiments. The data reduction package *APEX*4 v2021.10-0 was

used for data collecting and processing. Analysis of the integrated data did not show any decay. The data were corrected for systematic errors and absorption: Numerical absorption correction based on integration over a multifaceted crystal model and empirical absorption correction based on spherical harmonics according to the point group symmetry using equivalent reflections. The structures were solved by the direct methods using *SHELXT*-2018/2 [8] and refined by the full-matrix least-squares on F^2 using *SHELXL*-2018/3 [9]. Non-hydrogen atoms were refined anisotropically. The positions of the hydrogen atoms of the methyl group were found using a rotating group refinement with idealized tetrahedral angles. The other hydrogen atoms were inserted at the calculated positions and refined as riding atoms. The disorder, if present, was resolved using free variables and reasonable restraints on geometry and anisotropic displacement parameters. The unit cell of **5c** contained highly disordered solvent molecules, which were treated as a diffuse contribution to the overall scattering without specific atom positions by PLATON/SQUEEZE-70422 [10].

Deposition numbers 2176393 (for **5c**) and 2176394 (for **6c**) contain the supplementary crystallographic data for this paper. These data are provided free of charge by the joint Cambridge Crystallographic Data Centre and Fachinformationszentrum Karlsruhe Access Structures service www.ccdc.cam.ac.uk/structures.

Crystallographic data for 5c. Squeezed solvent info is not included in the formulae and related items such as molecular weights and calculated densities. $C_{21}H_{12}BrCl_3$, pale yellow plank (0.442 × 0.351 × 0.115 mm^3), formula weight 450.57 g mol^{-1}; monoclinic, $P2_1/c$ (No. 14), a = 12.8189(6) Å, b = 24.3901(12) Å, c = 7.2674(4) Å, β = 93.7357(13)°, V = 2267.4(2) Å^3, Z = 4, Z' = 1, T = 110(2) K, d_{calc} = 1.320 g cm^{-3}, μ(Mo $K\alpha$) = 2.167

mm^{-1}, F(000) = 896; $T_{max/min}$ = 0.6001/0.3120; 130265 reflections were collected (1.592° ≤ θ ≤ 27.877°, index ranges: –16 ≤ h ≤ 16, –32 ≤ k ≤ 32, and –9 ≤ l ≤ 9), 5413 of which were unique, R_{int} = 0.0537, R_σ = 0.0182; completeness to θ of 27.877° 100.0 %. The refinement of 236 parameters with no restraints converged to $R1$ = 0.0986 and $wR2$ = 0.3292 for 4772 reflections with $I > 2\sigma(I)$ and $R1$ = 0.1053 and $wR2$ = 0.3419 for all data with goodness-of-fit S = 1.076 and residual electron density $\rho_{max/min}$ = 4.207 and –1.285 e Å^{-3}, rms 0.248; max shift/e.s.d. in the last cycle 0.001.

Crystallographic data for 6c. $C_{47}H_{67}BrCl_3O_{3.50}P$, colorless prism (0.517 × 0.401 × 0.275 mm^3), formula weight 905.23 g mol^{-1}; monoclinic, $P2_1/c$ (No. 14), a = 29.762(2) Å, b = 17.6579(14) Å, c = 18.2966(14) Å, β = 97.4019(18)°, V = 9535.2(13) Å^3, Z = 8, Z' = 2, T = 110(2) K, d_{calc} = 1.261 g cm^{-3}, μ(Mo $K\alpha$) = 1.102 mm^{-1}, F(000) = 3824; $T_{max/min}$ = 0.5887/0.4196; 213317 reflections were collected (1.693° ≤ θ ≤ 26.733°, index ranges: –37 ≤ h ≤ 37, –22 ≤ k ≤ 22, and –23 ≤ l ≤ 23), 20128 of which were unique, R_{int} = 0.0680, R_σ = 0.0329; completeness to θ of 26.733° 99.4 %. The refinement of 1052 parameters with 154 restraints converged to $R1$ = 0.0556 and $wR2$ = 0.1392 for 16506 reflections with $I > 2\sigma(I)$ and $R1$ = 0.0697 and $wR2$ = 0.1464 for all data with goodness-of-fit S = 1.091 and residual electron density $\rho_{max/min}$ = 0.770 and –0.527 e Å^{-3}, rms 0.083; max shift/e.s.d. in the last cycle 0.002.

Materials

Starting complex [FeCp(η^6-$C_6H_5CH_3$)][PF_6] was prepared according to literature procedure [11].

1. Synthesis of bis(chlorophenyl)acetylenes 3a–c

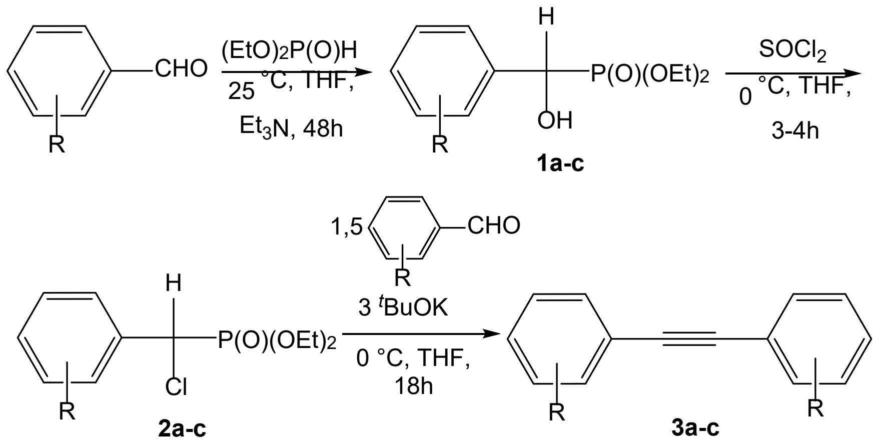

R = 2-Cl (a), 3-Cl (b), 4-Cl (c)

1.1. Synthesis of 1,2-bis(2-chlorophenyl)ethyne (3a). A mixture of 2-chlorobenzaldehyde (30 g, 0.21 mol), diethyl phosphite (29.5 g, 27.5 ml, 0.21 mol) and Et_3N (4 ml, 0.029 mol) in THF (150 ml) was stirred at 25 °C for 2 days to afford diethyl (hydroxy(aryl)methyl)phosphonate **1**. Afterwards, thionyl chloride (30.5 g, 18.6 ml, 0.26 mol) was added dropwise to the reaction mixture at 0 °C and it was stirred for 4 h, giving diethyl (chloro(aryl)methyl)phosphonate **2**. After that, excess of thionyl chloride was evaporated at reduced pressure. In a final step, to mixture of **2** and starting 2-chlorobenzaldehyde (30 g, 0.21 mol) in absolute THF (200 ml) under a nitrogen atmosphere, potassium *tert*-butoxide (50 g, 0.45 mol) was slowly added during 4 h at 0 °C. Afterwards, the reaction mixture stirred at 25 °C for 18 h. THF was evaporated in vacuum and the product was extracted by dichloromethane, washed with brine, the layers were separated and the organic layer was dried over $MgSO_4$. The organic layer was filtered, the solvent was evaporated on a rotary evaporator and in vacuum. The mixture was recrystallized from hot ethanol and the crystalline precipitate was washed with cold ethanol to give **3a** as pale white crystals (5.4 g, 10.4 %). M.p.: 81-82 °C. ^1H NMR (d_6-DMSO, 25 °C): δ (ppm) 7.26-7.37 (m, 4H, Ph), 7.38-7.45 (m, 4H, Ph). IR (KBr, cm^{-1}): 415 (m), 457 (m), 471 (w), 537 (w), 611(m), 696 (m), 721 (m), 752 (s, C-Cl), 800

(w), 839 (w), 860 (m), 882 (m), 945 (w), 1034 (m), 1053 (m), 1093 (w), 1125 (w), 1157 (w), 1213 (w), 1238 (w), 1273 (m), 1342 (m), 1445 (s), 1477(s), 1574 (w), 1596 (w), 1922 (w), 1954 (w), 2855 (w), 2928 (w), 3018 (w), 3066 (w).

Literature data for **3a** can be found in Reference [12].

1.2. Synthesis of 1,2-bis(3-chlorophenyl)ethyne (3b). A mixture of 3-chlorobenzaldehyde (30 g, 0.21 mol), diethyl phosphite (29.5 g, 27.5 ml, 0.21 mol) and Et_3N (4 ml, 0.029 mol) in THF (150 ml) was stirred at 25 °C for 2 days to afford diethyl (hydroxy(aryl)methyl)phosphonate **1**. Afterwards, thionyl chloride (30.5 g, 18.6 ml, 0.26 mol) was added dropwise to the reaction mixture at 0 °C and it was stirred for 4 h, giving diethyl (chloro(aryl)methyl)phosphonate **2**. After that, excess of thionyl chloride was evaporated at reduced pressure. In a final step, to mixture of **2** and starting 3-chlorobenzaldehyde (30 g, 0.21 mol) in dry THF (200 ml), potassium *tert*-butoxide (50 g, 0.45 mol) was slowly added during 4 h under a nitrogen atmosphere at 0 °C. Afterwards, the reaction mixture stirred at 25 °C for 18 h. THF was evaporated in vacuum. The reaction mixture was extracted by brine and dichloromethane, the layers were separated, and the organic layer was dried over $MgSO_4$. The organic layer was filtered, the solvent was evaporated on a rotary evaporator and in vacuum. The mixture was recrystallized from hot ethanol and the crystalline precipitate was washed with cold ethanol to give **3b** as pale white crystals (24.9 g, 48 %). M.p.: 83 °C. ^{1}H NMR (d_6-DMSO, 25 °C): δ (ppm) 7.30-7.32 (m, 2H, Ph), 7.36-7.42 (m, 8H, Ph). IR (KBr, cm^{-1}): 416 (m), 457 (m), 471 (w), 535 (w), 612 (m), 695 (m), 722 (m), 755 (s, C-Cl), 800 (w), 836 (w), 858 (m), 883 (m), 945 (w), 1031 (m), 1056 (m), 1096 (w), 1122 (w), 1156 (w), 1216 (w), 1245 (w), 1273 (m), 1341 (m), 1446 (s), 1476 (s), 1574 (w), 1597 (w), 1923 (w), 1955 (w), 2854 (w), 2927 (w), 3017 (w), 3065 (w).

Literature data for **3b** can be found in Reference [12].

1.3. Synthesis of 1,2-bis(4-chlorophenyl)ethyne (3c). A mixture of 4-chlorobenzaldehyde (30.05 g, 0.21 mol), diethyl phosphite (29.5 g, 27.5 ml, 0.21 mol) and Et_3N (4 ml, 0.029 mol) in THF (150 ml) was stirred at 25 °C for 2 days to afford diethyl (hydroxy(aryl)methyl)phosphonate **1**. Afterwards, thionyl chloride (30.5 g, 18.6 ml, 0.26 mol) was added dropwise to the reaction mixture at 0 °C, and it was stirred for 3 h, giving diethyl (chloro(aryl)methyl)phosphonate **2**. After that, excess of thionyl chloride was evaporated at reduced pressure. As a final step, to mixture of **2** and starting 4-chlorobenzaldehyde (30 g. 0.21 mol) in dry THF (200 ml), potassium *tert*-butoxide (50 g, 0.45 mol) was slowly added during 4 h under a nitrogen atmosphere at 0 °C. Afterwards, the reaction mixture stirred at 25 °C for 18 h. On the next day, THF was evaporated in vacuum. The reaction mixture was extracted by brine and dichloromethane, the layers were separated, and the organic layer was dried over $MgSO_4$. The organic layer was filtered, the solvent was evaporated on a rotary evaporator and in vacuum. The mixture was recrystallized from hot ethanol and the crystalline precipitate was washed with cold ethanol to give **3c** as pale yellow crystals (27.4 g, 52.9%). M.p.: 183-184 °C. ^1H NMR (d_6-DMSO, 25 °C): δ (ppm) 7.51 (d, 4H, Ph, $^3J_{HH}$ = 8.77 Hz), 7.59 (d, $^3J_{HH}$ = 8.77 Hz, 4H, Ph). IR (KBr, cm^{-1}): 475 (w), 514 (m), 655 (m), 825 (w), 832 (s, C-Cl), 847 (w), 1010 (m), 1052 (w), 1088 (s), 1117 (w), 1155 (w), 1259 (w), 1400 (w), 1492 (s), 1504 (w), 1593 (w), 1656 (w), 1911 (w), 2344 (w), 2364 (w).

Literature data for **3c** can be found in Reference [12].

2. Synthesis of chloro(dichloromethyl)benzenes 4a–c.

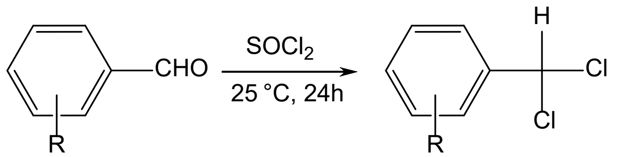

4a-c

R = 2-Cl (**a**), 3-Cl (**b**), 4-Cl (**c**)

2.1. Synthesis of 1-chloro-2-(dichloromethyl)benzene (4a). 2-Chlorobenzaldehyde (21.5 g, 0.15 mol) was slowly added to a mixture of thionyl chloride (229 g, 140 ml, 1.93 mol) and dimethylformamide (3 ml, 0.04 mol) during 30 min under a nitrogen atmosphere at 0 °C. Afterwards, the reaction mixture stirred at 25 °C for 24 h. On the next day, excess of $SOCl_2$ was evaporated at reduced pressure. Water was added to the remaining residue, and then the reaction mixture was extracted with Et_2O. The aqueous layer was again extracted with Et_2O, and the organic layers were collected and dried over $MgSO_4$. The reaction mixture was filtered over $MgSO_4$, Et_2O was evaporated, and the reaction residue was distilled in vacuum (11 mm Hg, bp = 92–93 °C) to give **4a** as pale colorless liquid (23.17 g, 96%). ^{1}H NMR (d_1-$CDCl_3$, 25 °C): δ (ppm) 7.23 (s, 1H, $CHCl_2$), 7.30-7.37 (m, 1H, Ph), 7.37-7.44 (m, 2H, Ph), 7.99 (m, 1H, Ph).

Literature data for **4a** can be found in Reference [13].

2.2. Synthesis of 1-chloro-3-(dichloromethyl)benzene (4b). 3-Chlorobenzaldehyde (21.5 g, 0.15 mol) was slowly added to a mixture of thionyl chloride (229 g, 140 ml, 1.93 mol) and dimethylformamide (3 ml, 0.04 mol) during 30 min under a nitrogen atmosphere at 0 °C. Afterwards, the reaction mixture stirred at 25 °C for 24 h. On the next day, excess $SOCl_2$ was evaporated at reduced pressure. Water was added to the remaining residue, and then the reaction mixture was extracted with Et_2O. The aqueous layer was again extracted with Et_2O, and the organic layers were collected and dried over $MgSO_4$. The reaction mixture was filtered over $MgSO_4$, Et_2O was evaporated, and

the reaction residue was distilled in vacuum (11 mm Hg, bp = 107–108 °C) to give **4b** as pale colorless liquid (21.96 g, 96%). ^{1}H NMR (d_1-$CDCl_3$, 25 °C): δ (ppm) 6.63 (s, 1H, $CHCl_2$), 7.30-7.57 (m, 4H, Ph).

Literature data for **4b** can be found in Reference [13].

2.3. Synthesis of 1-chloro-4-(dichloromethyl)benzene (4c). 4-Chlorobenzaldehyde (21.5 g, 0.15 mol) was slowly added to a mixture of thionyl chloride (229 g, 140 ml, 1.93 mol) and dimethylformamide (3 ml, 0.04 mol) during 4 h under a nitrogen atmosphere at 0 °C. Afterwards, the reaction mixture stirred at 25 °C for 15 h. On the next day, $SOCl_2$ was evaporated. The water was added to the remaining residue, and then the reaction mixture was extracted with Et_2O. The aqueous layer was extracted with Et_2O, and the organic layers were collected and dried over $MgSO_4$. The reaction mixture was filtered, Et_2O was evaporated, and the reaction mixture was distilled in vacuum (11 mm Hg, bp = 100–101 °C) to give **4c** as pale colourless liquid (21.29 g, 89%). ^{1}H NMR (d_1-$CDCl_3$, 25 °C): δ (ppm) 6.71 (s, 1H, $CHCl_2$), 7.38-7.43 (m, 2H, Ph), 7.52-7.57 (m, 2H, Ph).

Literature data for **4c** can be found in Reference [13].

3. Synthesis of 1,2,3-tris(chlorophenyl)cyclopropenyl bromides 5b,c.

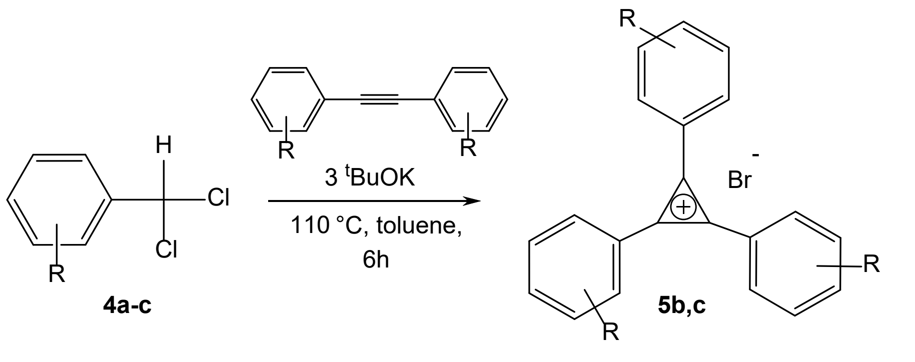

R = 3-Cl (**b**), 4-Cl (**c**)

3.1. Synthesis of 1,2,3-tris(3-chlorphenyl)cyclopropenyl bromide (5b). A mixture of 1-chloro-3-(dichloromethyl)benzene (**4b**, 10.58 g, 0.055 mol) and toluene (30 ml) was

added dropwise to the solution of 1,2-bis(2-chlorophenyl)ethyne (**3b**, 8.85 g, 0.036 mol) and *tert*-butoxide (10.31 g, 0.105 mol) in 250 mL of toluene to give a dark red dense reaction mixture. The reaction was carried out under a nitrogen atmosphere with intensive stirring. After adding the reagent, the reaction mixture was stirred at 100–110 °C for 6 h. In the next step, water (100 ml) was added to the reaction mixture. The toluene layer was separated, and the aqueous layer was extracted twice with Et_2O (2 × 50 ml). The organic layers were pooled and dried over $MgSO_4$. The concentrated aqueous solution of HBr (0.065 mol) was added dropwise to the solution with stirring at 25 °C. The precipitate of **5b** was filtered, dried and recrystallized from hot acetonitrile. The crystalline precipitate was washed with acetonitrile to give **5b** as pale yellow crystals (3.41 g, 21.6%). M.p.: 206°C. ^{1}H NMR (d_6-DMSO, 25°C): δ (ppm) 7.37-7.41 (m, 3H, Ph), 7.53-7.62 (m, 9H, Ph). ^{13}C NMR (d_6-DMSO, 25°C): δ (ppm) 128.2 (s, Ph), 129.8 (s, Ph), 131.6 (s, Ph), 131.9 (s, Ph), 133.4 (s, Ph), 135.3 (s, Ph), 144.7 (s, C_3-ring). IR (KBr, cm^{-1}): 408 (w), 436 (m), 467 (w), 520 (m), 532 (w), 696 (s), 724 (w), 795 (s, C-Cl), 847 (s), 878 (m), 996 (w), 1079 (w), 1140 (w), 1160 (w), 1273 (w), 1305 (w), 1398 (w), 1428 (m), 1464 (w), 1490 (s), 1566 (s), 1589 (s), 1728 (w), 1919 (w), 1987 (w).

3.2. Synthesis of 1,2,3-tris(4-chlorphenyl)cyclopropenyl bromide (5c). A mixture of 1-chloro-4-(dichloromethyl)benzene (**4c**, 21.17 g, 0.11 mol) and toluene (50 ml) was added dropwise to the solution of 1,2-bis(4-chlorophenyl)ethyne (**3c**, 17.69 g, 0.072 mol) and *tert*-butoxide (20.61 g, 0.21 mol) in 400 mL of toluene to give a dark red dense reaction mixture. The reaction was carried out under a nitrogen atmosphere with intensive stirring. After adding the reagent, the reaction mixture stirred at 100–110 °C for 6 h. In the next step, water (200 ml) was added to the reaction mixture. The toluene layer was separated, and the aqueous layer was extracted twice with diethyl ether (2 ×

100 ml). The organic layers were pooled and dried over $MgSO_4$. The concentrated aqueous solution of HBr (0.13 mol) was added dropwise to the solution with stirring at 25 °C. The precipitate of **5c** was filtered, dried, and recrystallized from hot acetonitrile. The crystalline precipitate was washed with acetonitrile to give **5c** as pale yellow crystals (4.58 g, 14.5%). M.p.: 238°C. ^{1}H NMR (d$_6$-DMSO, 25°C): δ (ppm) 7.54 (d, $^3J_{HH}$ = 8.3 Hz, 6H, Ph), 7.67 (d, $^3J_{HH}$ = 8.0 Hz, 6H, Ph). ^{13}C NMR (d$_6$-DMSO, 25°C): δ (ppm) 121.1 (s, Ph), 128.2 (s, Ph), 131.6 (s, Ph), 135.3 (s, Ph), 144.7 (s, C_3-ring). IR (KBr, cm^{-1}): 413 (s), 507 (s), 605 (w), 645 (w), 706 (w), 725 (w), 784 (w), 835 (s, C-Cl), 1006 (s), 1085 (s), 1107 (w), 1128 (w), 1174 (m), 1223 (w), 1294 (w), 1394 (s), 1414 (s), 1495 (s), 1533 (w), 1581 (s), 1924 (w), 2589 (w), 3031 (w), 3082 (w).

4. Synthesis of tributyl(1,2,3-tris(chlorophenyl)cyclopropenyl)phosphonium bromides 6b,c.

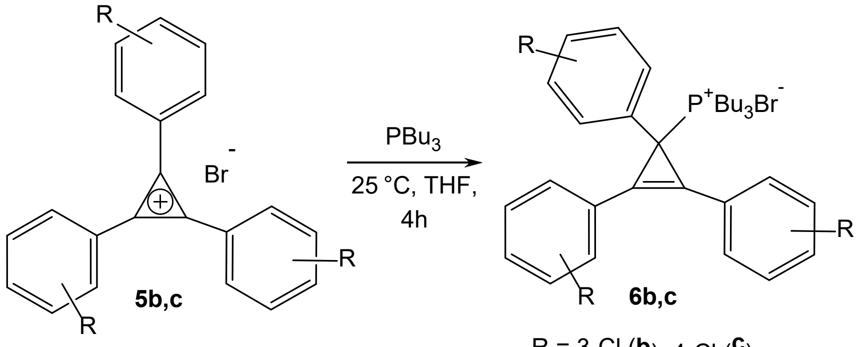

R = 3-Cl (**b**), 4-Cl (**c**)

4.1. Synthesis of tributyl(1,2,3-tris(3-chlorophenyl)cyclopropenyl)phosphonium bromide (6b). In a manner similar to Reference [14], a mixture of 1,2,3-tris(3-chlorophenyl)cyclopropenyl bromide (**5b**, 0.23 g, 0.51 mmol) and PBu_3 (0.1 g, 0.13 ml, 0.51 mmol) in THF (2.5 ml) was stirred at 25 °C for 4 h. Afterwards, the reaction mixture was kept at −18 °C for 5 days. Formed crystalline precipitate was collected by filtration and washed with cold THF to give tributyl(1,2,3-tris(3-chlorophenyl)cyclopropenyl)phosphonium bromide (**6b**) as colorless crystals (0.22 g, 38.6%). M.p.: 171 °C. ^{1}H NMR (d$_6$-DMSO, 25°C): δ (ppm) 0.73 (t, $^3J_{HH}$ = 7.0 Hz, 9H,

CH_3), 1.20-1.38 (m, 12H, CH_2), 2.31-2.4 (m, 6H, P-CH_2), 7.48-7.51 (m, 2H, Ph), 7.63 (s, 1H, Ph), 7.66-7.72 (m, 5H, Ph), 7.95-7.98 (m, 2H, Ph), 8.03-8.05 (m, 2H, Ph). ^{31}P NMR (d_6-DMSO, 25°C): δ (ppm) 39.6. ^{13}C NMR (d_6-DMSO, 25°C): δ (ppm) 13,9 (s, CH_3), 20.0 (s, CH_2), 20.5 (s, CH_2), 24.1 (d, $^1J_{PH}$ = 10.4 Hz, P-CH_2), 24.3 (br.s, P-C), 113.9 (s, C=C), 127.5 (d, $^2J_{HH}$ = 2.7 Hz, Ph), 128.4 (s, Ph), 128.9 (s, Ph), 129.8 (s, Ph), 129.9 (s, Ph), 130.0 (s, Ph), 130.1 (s, Ph), 132.4 (s, Ph), 132.7 (s, Ph), 135.0 (s, Ph), 135.4 (s, Ph). IR (KBr, cm^{-1}): 463 (w), 488 (w), 561 (w), 582 (w), 679 (s), 715 (m), 784 (s), 802 (s, C-Cl), 875 (m), 900 (w), 969 (w), 996 (w), 1056 (w), 1076 (s), 1098 (s), 1156 (m), 1226 (m), 1258 (m), 1291 (m), 1346 (w), 1382 (w), 1409 (s), 1463 (s), 1561 (s), 1589 (s), 1846 (s), 2793 (w), 2870 (m), 2931 (m), 2959 (m), 3051 (w).

4.2. Synthesis of tributyl(1,2,3-tris(4-chlorophenyl)cyclopropenyl)phosphonium bromide (6c). In a manner similar to Reference [14], a mixture of 1,2,3-tris(4-chlorophenyl)cyclopropenyl bromide (**5c**, 0.5 g, 1.1 mmol) and PBu_3 (0.22 g, 0.27 ml, 1.1 mmol) in THF (5 ml) was stirred at 25 °C for 4–5 h. Afterwards, the reaction mixture was kept at −18 °C for 5 days. Formed crystalline precipitate was collected by filtration and washed with cold THF to give tributyl(1,2,3-tris(4-chlorophenyl)cyclopropenyl)phosphonium bromide (**6c**) as colorless crystals (0.20 g, 34.1%). M.p.: 182°C. ^{1}H NMR (d_6-DMSO, 25°C): δ (ppm) 0.73 (t, $^3J_{HH}$ = 7.0 Hz, 9H, CH_3), 1.20-1.38 (m, 12H, CH_2), 2.26-2.37 (m, 6H, CH_2), 7.48 (d, $^3J_{HH}$ = 8.5 Hz, 2H, Ph), 7.65 (d, $^3J_{HH}$ = 8.5 Hz, 2H, Ph), 7.71 (d, $^3J_{HH}$ = 8.5 Hz, 4H, Ph), 8.00 (d, $^3J_{HH}$ = 8.5 Hz, 4H, Ph). ^{31}P NMR (d_6-DMSO, 25°C): δ (ppm) 39.6. ^{13}C NMR (d_6-DMSO, 25°C): δ (ppm) 13,9 (s, CH_3), 20.1 (s, CH_2), 20.6 (s, CH_2), 24.2 (d, $^1J_{PH}$ = 11.7 Hz, P-CH_2), 24.3 (br.s, P-C), 113.3 (s, C=C), 124.5 (s, Ph), 130.5 (s, Ph), 130.9 (s, Ph), 131.4 (s, Ph), 132.7 (s, Ph), 134.4 (s, Ph), 136.9 (s, Ph), 1371 (s, Ph). IR (KBr, cm^{-1}): 514 (m), 599 (w), 644 (w), 725 (w), 764 (w), 833 (s), 938 (w), 968 (w), 1005 (m), 1088 (s), 1181 (w), 1222 (w),

1275 (w), 1296 (w), 1397 (m), 1464 (w), 1489 (s), 1561 (w), 1590 (m), 1817 (w), 2872 (m), 2931 (m), 2959 (m), 3017 (w).

5. Synthesis of sodium bis(diglyme) 3,4,5-tris(chlorophenyl)-1,2-diphosphacyclopentadienides 7.

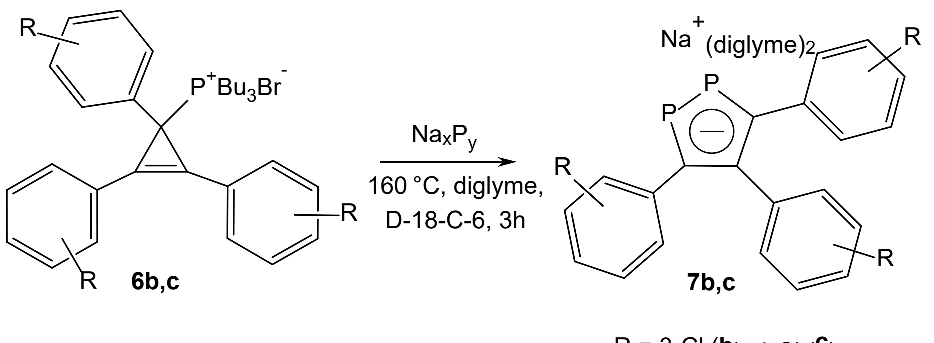

R = 3-Cl (**b**), 4-Cl (**c**)

5.1. Sodium bis(diglyme) 3,4,5-tris(3-chlorophenyl)-1,2-diphosphacyclopentadienide (7b). A mixture of 0.04 g Na (1.7 mmol) and 0.1 g P_4 (0.8 mmol) in diglyme (10 mL) was refluxed for 6 h in the presence of catalytic amounts of dibenzo-18-crown-6 to give a mixture of sodium polyphosphides. The reaction mixture was cooled to room temperature and 0.3 g (0.7 mmol) **6b** was added, and the mixture was refluxed for an additional 3 h. The reaction mixture was filtered, the solvent was evaporated in vacuum, and the remaining residue was washed three times with *n*-hexane (20 mL) and dried in vacuum, resulting in 0.29 g (60%) of **7b** as a red brown powder. ^{1}H NMR (d_8-THF): δ = 3.16 (s, 12H, MeO), 3.28 (t, $^3J_{HH}$ = 5.3 Hz, 8H, OCH_2), 3.34 (t, $^3J_{HH}$ = 5.3 Hz, 8H, OCH_2), 7.38-7.42 (m, 3H, Ph), 7.54-7.68 (m, 5H, Ph), 7.71-7.82 (m, 2H, Ph), 7.93-8.01 (m, 2H, Ph). ^{31}P NMR (d_8-THF): δ = 200.7 (s). ^{13}C NMR (d_8-THF): δ = 56.1 (s, MeO), 68.0 (s, OCH_2), 69.5 (s, OCH_2), 128.3 (s, Ph), 129.0 (s, Ph), 129.7 (s, Ph), 129.9 (s, Ph), 130.0 (s, Ph), 130.3 (s, Ph), 132.5 (s, Ph), 132.8 (s, Ph), 134.6 (s, Ph), 135.2 (s, Ph), 142.6 (m, C_{ipso}), 146.1 (t, $^2J_{PC}$ = 10.2 Hz, C_3P_2), 155.9 (m, C_3P_2).

5.2. Synthesis of sodium 3,4,5-tris(4-chlorophenyl)-1,2-diphosphacyclopentadienide (7c). A mixture of 0.92 g Na (40 mmol) and 2.5 g P_4 (20 mmol) in diglyme (50 mL) was refluxed for 6 h in the presence of 0.072 g dibenzo-18-crown-6 to give a mixture of sodium polyphosphides. The reaction mixture was cooled to room temperature, 14.25 g (20 mmol) **6b** was added, and the mixture was refluxed for an additional 3 h. The reaction mixture was filtered, the solvent was evaporated in vacuum, and the remaining residue was washed three times with *n*-hexane (20 mL) and dried, resulting in 9.12 g (63%) of **7c** as a red brown powder. ^{1}H and ^{31}P NMR spectroscopic data were in agreement with the literature [15].

6. Synthesis of 3,4,5-tris(chlorophenyl)-1,2-diphosphaferrocenes 8.

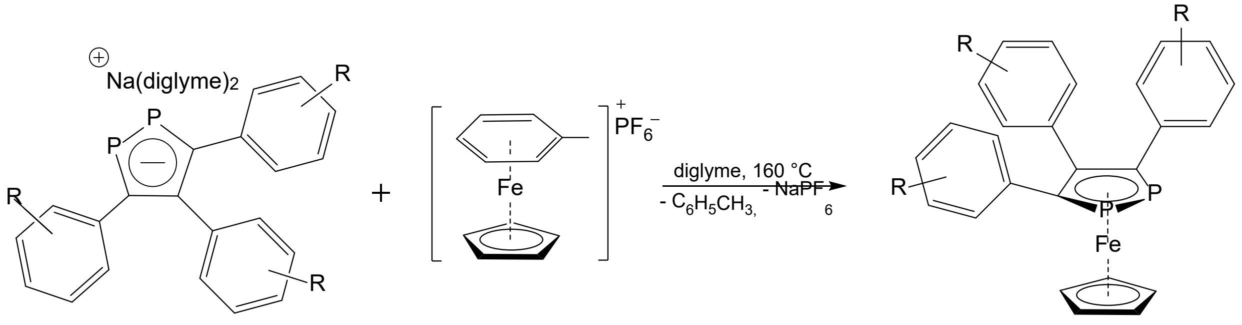

7b,c

R = 3-Cl(b), 4-Cl(c)

8b,c

6.1. Synthesis of 3,4,5-tris(3-chlorophenyl)-1,2-diphosphaferrocene (8b): [FeCp(η^6-$C_6H_5CH_3$)][PF_6] (0.29 g, 0.3 mmol) was added to sodium 3,4,5-tris(3-chlorophenyl)-1,2-diphosphacyclopentadienide (**7b**, 0.29 g, 0.3 mmol), which was dissolved in 10 mL diglyme and cooled to −80 °C. The reaction mixture was stirred at low temperature for 3 h and then heated to 160 °C for an additional 3 h. Then, the reaction mixture was cooled to room temperature, the solvent was evaporated, and the remaining solid was dissolved in 30 mL toluene, passed through a layer of silica (4–5 cm), and the silica was additionally washed with toluene (3 × 15 ml). After removal of the solvent, **8b** was obtained as a reddish powder (0.18 g, 68% yield). ^{1}H NMR ($CDCl_3$, δ, ppm): 4.61 (s, 5H, C_p), 6.88-6.93 (m, 3H, Ph), 7.02-7.60 (m, 4H, Ph), 7.14-7.21 (m, 2H, Ph), 7.33-7.42

(m, 3H, Ph). $^{31}P\{^1H\}$ NMR ($CDCl_3$, δ, ppm): -10.2 (s). $^{13}C\{^1H\}$ ($CDCl_3$, δ, ppm, J, Hz): 74.9 (s, C_p), 105.8 (ps.t, $^2J_{PC}$ = 6.8, P_2C_3), 116.5 (ps.t, $^1J_{PC}$ = 43.1, P_2C_3), 128.4 (s, Ph), 129.1 (s, Ph), 129.5 (s, Ph), 130.2 (s, Ph), 130.5 (s, Ph), 131.6 (s, Ph), 133.7 (s, Ph), 134.8 (s, Ph), 135.1 (s, Ph), 137.6 (s, Ph), 143.6 (m, Ph). IR (KBr, cm^{-1}): 3048 (w), 2945 (w), 2917 (w), 2856 (w), 1896 (w), 1487 (s), 1396 (m), 1262 (w), 1174 (w), 1087 (s), 1012 (s), 964 (w), 933 (w), 828 (s), 816 (s), 743 (s), 720 (w), 653 (w), 649 (w), 556 (m), 522 (w), 514 (w), 475 (w), 452 (w).

The synthesis of 3,4,5-tris(4-chlorophenyl)-1,2-diphosphaferrocene (**8c**) has previously been reported [6].

References

1. M.J. Frisch, G.W. Trucks, H.B. Schlegel, G.E. Scuseria, M.A. Robb, J.R. Cheeseman, G. Scalmani, V. Barone, G.A. Petersson, H. Nakatsuji, et al., Gaussian 09 Revision A.02; Gaussian, Inc.: Wallingford, CT, USA, **2016**.

2. C. Adamo and V. Barone, Toward reliable density functional methods without adjustable parameters: The PBE0 model, *J. Chem. Phys.*, **1999**, *110*, 6158–6170.

3. F. Weigend and R. Ahlrichs, Balanced basis sets of split valence, triple zeta valence and quadruple zeta valence quality for H to Rn: Design and assessment of accuracy, *Phys. Chem. Chem. Phys.*, **2005**, *7*, 3297–3305.

4. S. Grimme, J. Antony, S. Ehrlich and H.A. Krieg, A consistent and accurate ab initio parametrization of density functional dispersion correction (DFT-D) for the 94 elements H-Pu, *J. Chem. Phys.*, **2010**, *132*, 154104–154119.

5. T. Yanai, D. Tew and N. Handy, A new hybrid exchange–correlation functional using the Coulomb-attenuating method (CAM-B3LYP), *Chem. Phys. Lett.*, **2004**, *393*, 51–57.

6. Bezkishko, I.A.; Zagidullin, A.A.; Khrizanforov, M.N.; Gerasimova, T.P.; Ivshin, K.A.; Kataeva, O.N.; Ganushevich, Y.S.; Miluykov, V.A.; Lönnecke, P.; Hey-Hawkins, E. *Inorg. Chem. Front.* **2022**, *9,* 2608-2616.

7. M. N. Khrizanforov, D. M. Arkhipova, R. P. Shekurov, T. P. Gerasimova, V. V. Ermolaev, D. R. Islamov, V. A. Miluykov, O. N. Kataeva, V. V. Khrizanforova, O. G. Sinyashin and Y. H. Budnikova, J. Solid State Electrochem., **2015**, *19*, 2883–2890.

8. G. M. Sheldrick, *SHELXT* – Integrated space-group and crystal-structure determination, *Acta Crystallogr., Sect. A: Found. Adv.* **2015**, *71*, 3–8.

9. G. M. Sheldrick, Crystal structure refinement with *SHELXL*, *Acta Crystallogr., Sect. C: Struct. Chem.* **2015**, *71*, 3–8.

10. A. L. Spek, *PLATON* SQUEEZE: a tool for the calculation of the disordered solvent contribution to the calculated structure factors, *Acta Crystallogr., Sect. C: Struct. Chem.* **2015**, *71*, 9−18.

11. E. W. Abel and G. Wilkinson, *J. Chem. Soc.,* **1959**, 1501–1505.

12. M. J. Mio, L. C. Kopel, J. B. Braun, T. L. Gadzikwa, K. L. Hull, R. G. Brisbois, C.J. Markworth, and P.A. Grieco. *Org. Lett.* **2002**, *4*, 19, 3199–3202.

13. Wu, Jiang Zhou, Junpeng Shi, Yalei Zhu, Jintao, *Chin. J. Org. Chem*. **2016**, *36*, 1958−1962.

14. Zagidullin, A.A.; Khrizanforov, M.N.; Bezkishko, I.A.; Lönnecke, P.; Hey-Hawkins, E.; Miluykov, V.A. *J. Organomet. Chem.* **2021**, *956*, 122122.

15. Bezkishko I., Miluykov V., Sinyashin O., and Hey-Hawkins, E. *Phosphorus, Sulfur, Silicone and Rel. El.* **2011**, *186: 4*, 657−659.
